# The Effects of Injury Preventive Warm-Up Programs on Knee Strength Ratio in Young Male Professional Soccer Players

**DOI:** 10.1371/journal.pone.0050979

**Published:** 2012-12-03

**Authors:** Abdolhamid Daneshjoo, Abdul Halim Mokhtar, Nader Rahnama, Ashril Yusof

**Affiliations:** 1 Sports Centre, University of Malaya, Kuala Lumpur, Malaysia; 2 Faculty of Medicine, University of Malaya, Kuala Lumpur, Malaysia; 3 Faculty of Physical Education and Sport Science, University of Isfahan, Isfahan, Iran; Universidad Europea de Madrid, Spain

## Abstract

**Purpose:**

We aimed to investigate the effect of FIFA 11+ (11+) and HarmoKnee injury preventive warm-up programs on conventional strength ratio (CSR), dynamic control ratio (DCR) and fast/slow speed ratio (FSR) in young male professional soccer players. These ratios are related to the risk of injury to the knee in soccer players.

**Methods:**

Thirty-six players were divided into 3 groups; FIFA 11+, HarmoKnee and control (n = 12 per group). These exercises were performed 3 times per week for 2 months (24 sessions). The CSR, DCR and FSR were measured before and after the intervention.

**Results:**

After training, the CSR and DCR of knee muscles in both groups were found to be lower than the published normal values (0.61, 0.72, and 0.78 during 60°.s^−1^, 180°.s^−1^ and 300°.s^−1^, respectively). The CSR (60°.s^−1^) increased by 8% and FSR in the quadriceps of the non-dominant leg by 8% in the 11+. Meanwhile, the DCR in the dominant and non-dominant legs were reduced by 40% and 30% respectively in the 11+. The CSR (60°.s^−1^) in the non-dominant leg showed significant differences between the 11+, HarmoKnee and control groups (p = 0.02). As for the DCR analysis between groups, there were significant differences in the non-dominant leg between both programs with the control group (p = 0.04). For FSR no significant changes were found between groups.

**Conclusions:**

It can be concluded that the 11+ improved CSR and FSR, but the HarmoKnee program did not demonstrate improvement. We suggest adding more training elements to the HarmoKnee program that aimed to enhance hamstring strength (CSR, DCR and FSR). Professional soccer players have higher predisposition of getting knee injuries because hamstring to quadriceps ratio were found to be lower than the average values. It seems that the 11+ have potentials to improve CSR and FSR as well as prevent knee injuries in soccer players.

## Introduction

Most injuries in soccer occur in the lower extremities, especially the knee and ankle. They make up 17% of all injuries, i.e. the most common injury sites [1]. Majewski et al. [2] investigated 17,397 patients with 19,530 sport injuries over a 10-year period and showed that of 6434 players, over a third (37%) had 7769 injuries (39.8%) related to the knee. Soccer (35%) and skiing (26%) were shown to be the most viable sports contracting injuries, and majority of the injured players were men (68.1%) [2]. In addition, according to FIFA (2006) about 69.6% of the registered players are male. Hence, isokinetic studies on the knee muscles in the young male soccer players are substantial. They may help us to improve injury prevention [3].

One of the determining factors of knee injuries is the muscle balance between hamstring and quadriceps [4]–[7]. Low hamstring to quadriceps ratio is associated significantly with knee muscle injury. This strength ratio is acceptable to be between 0.5 to 0.8 depending on the angular velocity of the performed movement with the hamstring at least half as strong as the quadriceps [8], [9]. According to Grygorowicz et al. [9], one can estimate the risk of injury on the basis of the Hcon/Qcon ratio value [9]. Several researches have associated Conventional Strength Ratio (CSR) of below 0.61, 0.72, 0.78 during 60°.s^−1^, 180°.s^−1^ and 300°.s^−1^, respectively, with increased risk of injuries [7], [8], [10], [11]. The higher the value of the Hcon/Qcon ratio, the better the functional capability of the hamstring to stabilize the knee joint. Increased knee joint stability can prevent and decrease the risk of knee injury [9].

In addition to the CSR, a few researchers have suggested another method to evaluate knee joint condition which is referred as Dynamic Control Ratio (DCR) [6], [12]. DCR is calculated as peak net eccentric hamstring torque divided by peak net concentric quadriceps torque. The normal value of DCR above 1.00 at medium velocity was found to provide dynamic joint stability and reduce injuries [5], [7], [8], [10]. The anterior shear forces produced by the resistance at the attachment site of the limb to the lever arm in relation to the rotatory force of the maximally contracting quadriceps are thought to be counteracted by the eccentric contraction of the hamstrings. Therefore, the DCR is a useful strength ratio of the joint stabilizing effect on the hamstring muscles during knee extension [12].

Currently the two widely used preventive programs in soccer are the 11+ and HarmoKnee. FIFA introduced the 11+ which is an advanced version of the 11 program for prevention of lower extremity injuries. Soligard et al. [13] in a study showed that the 11+ was effective in reducing the incidence of injury in young female soccer players [13]. Brito et al. [14] conducted the 11+ program on young male soccer players and found that the 11+ program significantly increased the CSR at 60°.s^−1^ by 14.8% and the DCR by 13.8% only in the non-dominant leg [14]. Kiani et al. [15] later designed an exercise program called the HarmoKnee especially for prevention of knee injuries in soccer players [15]. The results showed that the intervention group was associated with a 77% decrease in knee injuries. Furthermore, it is shown that the most effective way to prevent injuries in young soccer players is to have a proper warm-up program [16]. The 11+ and HarmoKnee programs have some similarity in components such as running, forward and backward jump, walking lunge etc. [13], [15]. In addition, these programs have differences in training components. For example, the 11+ programs included elements such as Nordic hamstring, sideway bench, hip out, and hip in, circling partner, shoulder contact, bounding and plant & cut [13] while the HarmoKnee program contains elements such as hamstring curl, sit-up, bridging, activation muscles, defensive pressure technique [15]. The two programs are well incorporated in the warm-up of players so that they ensure compliance/consistency.

To our knowledge, there is no detailed research that compares the effect of the FIFA 11+ and HarmoKnee programs. There is not enough information that shows which program is more effective in injury prevention for young male soccer players. Moreover, knee injuries lead to long-term disability and impose enormous costs on teams and players. Identifying knee risk factors and injury prevention factors in the largest sport population in the world is a critical issue. Therefore, the aim of this study is to investigate the effects of the two programs on the CSR, DCR and FSR in young professional male soccer players.

## Methods

### Ethics Statement

All the participants were informed orally about the procedures they would undergo and their written consent was taken. Moreover, we obtain informed consent from the next of kin, caretakers or guardians on the behalf of the minors/child participants involved in the present study. The study was approved by the ethical committee of the Institute of Research Management and Monitoring, University of Malaya and the Sports Centre Research Committee.

### Participants

Thirty-six young male professional soccer players were selected as the participants of the present study. Their ages ranged from 17 to 20 years and they had all experienced playing soccer at a professional level. These players were employed by their clubs to participate in the national league. The clubs had almost daily training and played one match per week in a season. Players with history of major lower limb injury or disease were excluded from the study. Three professional teams were selected for this study (Table 1).

**Table 1:** Stature characteristics of the subjects (values are mean±SD).

Groups	11+ (n = 12)	HarmoKnee (n = 12)	Control (n = 12)
Age(y)	19.2(0.9)	17.7(0.4)	19.7(1.6)
Height(m)	1.81(5.1)	1.79(6.4)	1.83(4.6)
Mass(kg)	71.7(4.6)	71(7.6)	76.4(5.8)

### Procedure

At the mid- season of 2011, the coaches and team managers from the three professional teams were invited to a four-hour instruction course which aimed to introduce the intervention programs. Three under-21 (U21) teams from two professional soccer clubs (i.e., the Foolad Mobarakeh Sepahan Sport and Cultural Club, and the Esfahan Zob Ahan Cultural and Sport Club) volunteered to participate in this study. The players from one team were randomly selected and assigned to one of the intervention programs. Each team has about 30 professional players (matched), and from these 12 players were randomly picked to participate in the study. Each subjects performed only one of the selected warm-up programs. The groups were matched during pre-test using the knee strength measurements. One-way ANOVA did not show significant difference in pre-test between the 11+, HarmoKnee and control groups at all angular velocities of quadriceps and hamstrings (p>0.05).

Before starting the intervention programs, all the players attended a workshop to prescribe proper ways to carry out the exercises. This workshop was conducted for each team separately. None of the team knew about the exercises the other teams carried out. They all received video instructions and illustrations on the intervention programs. All the training sessions were supervised by one of the researchers to ensure their compliance with the programs. Verbal encouragements were given throughout the training period to help the participants concentrate on the quality of their movements. This research has been carried out in accordance with the ethical standards. We encouraged all subjects to maintain similar eating habits and sleeping patterns.

The players were coached on how to perform the exercises correctly. They also participated in familiarization sessions with the isokinetic machine. During these sessions, the subjects were fitted on the isokinetic system for a knee extension and flexion protocol. The settings were recorded to ensure the same positioning for all experimental tests. The concentric knee extension and flexion were monitored and where necessary corrected to ensure that they were fully familiar with the test protocol. The exercise prevention programs started on 15^th^ April, 2011 and completed on 15^th^ June, 2011 (24 sessions). The programs were performed as warm-ups before the general trainings.

### The Prevention Programs

#### The “11+” program

The 11+ consists of three parts, beginning with running exercises (part I), followed by six exercises to develop strength, balance, muscle control and core stability (part II), and ending with advanced running exercises (part III). The 11+ takes approximately 20–25 minutes to complete and replaces the usual warm-up before training. All exercises focus on core stability, neuromuscular control, eccentric hamstrings strength and agility (Table 2). These exercises were performed three times per week [13].

**Table 2:** The FIFA 11+. Exercises, duration and intensities of the structured warm-up program used (F-MARC).

Exercise	Duration
**Part 1: Running**	**8 minutes**
Straight ahead, hip out, hip in, circling partner, shoulder contact, quick forward & backwards (6 running items, each item 2 sets)	
**Part 2: strength, plyometric and balance**	**10 minutes**
** The bench:** Static, alternate legs and one leg lift and hold (3 items, each item 3 sets)	
** Sideways bench:** Static, raise & lower hip, with leg lift (3 items, 3 sets on each side)	
** Hamstring:** Beginner (3–5 repetition, 1 set), intermediate (7–10 repetition, 1 set), advanced (12–15 repetition, 1 set). (3 items)	
** Single-leg stance:** Hold the ball, throw the ball to a partner, test your partner (3 items, each item 2 sets)	
** Squats:** With toe raise, walking lunges, one-leg squats (3 items, each item 2 sets)	
** Jumping:** Vertical jumps, lateral jumps, box jumps (3 items, each item 2 sets)	
**Part 3: running exercise**	**2 minutes**
Across the pitch, bounding, plant & cut (3 items, each item 2 sets)	

#### The HarmoKnee program

The HarmoKnee prevention program was designed by Kiani et al. [15]. The aim of this prevention program is to increase overall awareness of injury risk, to provide a structured warm-up protocol, and to increase strength. Reportedly, the program can improve movement pattern and reduce knee injuries [15]. The training protocol consists of five parts: warm up, muscle activation, balance, strength and core stability, all of which can be combined and performed in a regular soccer training session. All the exercises are described in Table 3. The total program duration was 20 to 25 minutes. Similarly to the 11+, the HarmoKnee was also performed three times per week [15].

**Table 3:** The HarmoKnee training program. Exercises and duration of the structured warm-up program used.

Exercise	Duration
**Warm-up**	**≥10 min**
Jogging (≥4–6 min), Backward jogging on the toes (Approximately 1 min), High-knee skipping (Approximately 30 s),Defensive pressure technique (Approximately 30 s), One and one (≥2 min)	
**Muscle activation**	**Approximately 2 min**
Activation of calf muscles, quadriceps muscles, hamstring muscles, hip flexor muscles, groin muscles, hip andlower back muscles (6 item, each item 4 s for each leg/side)	
**Balance**	**Approximately 2 min**
Forward and backward double leg jumps, Lateral single leg jumps, Forward and backward single leg jumps,Double leg jump with or without ball (optional), (4 items, each item approximately 30 s)	
**Strength**	**Approximately 4 min**
Walking lunges in place, Hamstring curl (in pairs), Single-knee squat with toe raises (3 item, each itemApproximately 1 min)	
**Core stability**	**Approximately 3 min**
Sit-ups, Plank on elbows and toes, Bridging (3 items, each item approximately 1 min)	

#### Control group

For comparison, the control group was asked to continue with their regular team training and warm-up without any restrictions. In addition, before the commencement of the study, the control group was promised that they would receive the intervention program 8 weeks later. All groups participated in common training which consisted of technical and tactical drills such as; passing, shooting, dribbling and heading drills. In addition, they play in small-sided games such as; 5×5 m and 10×10 m square grids. Players in the control group were monitored closely not to perform any specific exercises that would contaminate our results.

### Isokinetic Test

A Biodex Isokinetic Dynamometer (Biodex 3, New York, USA) was used to assess the hamstring and quadriceps strength of the subjects. The Biodex System 3 has been shown to be a reliable instrument with high ICC (intraclass correlation coefficient) values (>0.90) for collecting isokinetic peak torque data in humans [17]–[19]. The strength measures recorded on an isokinetic dynamometer indicate the net moment generated at the joint minus the co-contraction of antagonist muscles and by passive structures of the joints at the end of the range of motion [6], [20]. All tests were carried out between 8 am and 11 am. Before each testing session, the dynamometer was gravitationally corrected in accordance with the manufacturer’s recommendations. The subjects performed a general cardiovascular warm-up for at least 5 min on a Monark cycle ergometer at a moderate pace (50–100 W) and followed by a 10-min dynamic stretching concentrating on the lower body [5].

Each subject was seated on the chair and assumed his most comfortable position to perform the best tests. Then the subject was secured with snug straps across the shoulder, chest and hip. The cuff of the dynamometer’s lever arm was attached proximal to the lateral malleolus of the ankle. Dynamometer orientation was fixed at 90° and tilted at 0°, while the seat orientation was fixed at 90° and the seatback tilted at 70–85°. The rotational axis of the knee joint was aligned with the dynamometer rotational axis. Device set-up and subject positioning were as per the manufacturer’s guidelines (Biodex system 3) which were also similar to previous researches [14], [21], [22]. The subject was instructed to complete 3 trials; two sub-maximal efforts and one maximal effort on the isokinetic machine. The subject then performed knee extension and flexion 3 times at each selected angular velocity with 5-s rest interval in between. They were also given a 1-min rest between different angular velocities and a 3-min break when machine setting was changed for the opposite leg. The order of testing was randomized for the preferred and non-preferred legs. The preferred leg is defined as the leg that the soccer player favours for kicking the ball. Encouragements by verbal coaching and visual feedback were given to all subjects. The isokinetic measurement was standardized according to the Biodex manufacturer’s manual (Biodex system 3), and similar to previous researches [5], [23]–[25]. Concentric isokinetic contractions were performed on the dynamometer at the speed of 60°.s^−1^ (low velocity), 180°.s^−1^ (medium velocity) and 300°.s^−1^ (high velocity), through a knee range of motion of 0 (flexed) to 90° (full extension). The order of testing for the different angular velocities (60°.s^−1^,180°.s^−1^ and 300°.s^−1^) was standardized from the slowest to the highest as recommended by Wilhite et al. [26]. The DCR was evaluated at 120°.s^−1^ (peak net eccentric hamstrings torque at 120°.s^−1^ divided by peak net concentric quadriceps torque at 120°.s^−1^). These testing speeds have been widely used for isokinetic muscle strength assessment in soccer players [27]–[29]. Selecting low (60°.s^−1^), medium (180°.s^−1^) and high (300°.s^−1^) isokinetic testing speeds are essential for optimal strength evaluation in bilateral mode, given that at low velocity, majority of muscle motor units are recruited, while higher velocity can enrich the force-velocity spectrum of the acting muscles [27]. Meanwhile, the hamstring and quadriceps strength measurements were performed twice. The pre-test was conducted one week prior to the first day of training and the post-test eight weeks later (three days after the last training session). All tests were conducted in the same order for each player at pre- and post-tests [5], [30]. Isokinetic testing was performed by a different member of the researcher team. The tester was blinded to the type of intervention the players participated in. Fast/slow speed ratio (FSR) of quadriceps and hamstrings before and after the interventions were calculated by net muscles peak torque at 300°.s^−1^ divided by the net muscles peak torque at 60°.s^−1^. The FSR was used as a variable because it is an indicator of how basic strength is maintained as angular velocity increases [25]. Net muscles peak torque was taken as the maximum value achieved during the 3 contractions.

### Statistical Analysis

The SPSS software (Version 18) was used for all analyses. For comparing the isokinetic strength in pre-test and post-test between groups (11+ vs HarmoKnee vs control), the one-way analysis of covariance (ANCOVA) was used with the pre-test values as covariate following the method of Pallant [31]. For assessing the isokinetic strength in every group (comparison of pre-test and post-test), the paired *t*-test was used. In addition, the delineation of leg dominance was analysed accordingly. In case of statistical significance, the post-hoc Bonferroni test was conducted. The Levene's test and Kolmogorov-Smirnov (KS) were employed for assessing homogeneity of variance among groups and normality of the distribution of scores (p>0.05). The data met the assumptions for linearity by using scatterplot (R squared value). The interaction between the covariate and the factor was used to test homogeneity of regression effect in covariate (p>0.05). A significant level was accepted at the 95% confidence level for all statistical parameters.

## Results

### CSR between pre- and Post-tests

The means of all isokinetic strength ratios are presented in Table 4. The CSR in the experimental groups at 60°.s^−1^ and 180°.s^−1^ angular velocities showed a higher trend in post-tests than pre-tests. In the 11+ group, paired *t*-test showed significant increase by 8% in post-test as compared with pre-test in the non-dominant leg at 60°.s^−1^ (t = 3.08, p = 0.01). In the HarmoKnee group, although the results showed a higher tendency in the post-test, they were of no significance (p>0.05).

**Table 4:** Conventional and dynamic strength ratio in dominant and non-dominant legs (values are mean±SD), and percentage of change (Δ) {values are mean (95% CI)} from pre- to post-test.

	Dominant leg			Non-dominant leg		
	Pre-test	Post-test	Δ% (95%CI)	Pre-test	Post-test	Δ% (95%CI)
**The 11+**						
**CSR**						
H/Q Con 60°.s^−1^	0.53±0.8	0.57±0.9	0.04(−0.1 to 0.2)	0.50±0.1	0.57±0.08	−0.08(−0.13 to−0.02)**
H/Q Con 180°.s^−1^	0.54±0.9	0.61±0.1	0.7(−0.001 to 0.13)	0.56±0.1	0.60±0.09	−0.04(−0.1 to 0.02)
H/Q Con 300°.s^−1^	0.72±0.2	0.73±0.2	0.005(−0.13 to 0.14)	0.75±0.2	0.74±0.2	0.01(−0.12 to 0.14)
**FSR**						
F/S Quadriceps	0.45±0.1	0.53±0.1	−1.08 (−0.16 to 0.004)	0.45±2	0.54±0.1	−0.08(−0.16 to −0.01)*
F/S Hamstring	0.60±0.2	0.67±0.2	−0.07(−0.18 to 0.3)	0.68±0.2	0.69±0.1	−0.01(−0.17 to 0.15)
**DCR**						
H_ECC_/Q_CON_ 120°.s^−1^	0.86±0.5	0.47±0.1	−0.4(0.1 to 0.7)*	0.82±0.4	0.48±0.1	−0.3(0.1 to 0.5)**
**HarmoKnee**						
**CSR**						
H/Q Con 60°.s^−1^	0.48±0.1	0.55±0.08	0.06(−0.15 to 0.02)	0.49±0.2	0.50±0.06	0.01(−0.11 to 0.08)
H/Q Con 180°.s^−1^	0.51±0.1	0.59±0.1	0.08(−0.17 to 0.01)	0.61±0.2	0.58±0.1	−0.04(−0.09 to 0.17)
H/Q Con 300°.s^−1^	0.83±0.2	0.72±0.1	−0.1(−0.05 to 0.3)	0.81±0.2	0.73±0.1	−0.08(−0.04 to 0.2)
**FSR**						
F/S Quadriceps	0.46±0.1	0.51±0.1	−0.06(−0.14 to 0.02)	0.48±0.1	0.51±0.1	−0.03(−0.09 to 0.03)
F/S Hamstring	0.79±0.2	0.68±0.1	0.1(−0.02 to 0.2)	0.79±0.2	0.72±0.1	0.07(−0.06 to 0.2)
**DCR**						
H_ECC_/Q_CON_ 120°.s^−1^	0.66±0.2	0.55±0.1	−0.1(−0.04 to 0.3)	0.72±0.4	0.51±0.1	0.2(−0.01 to 0.4)
**Control group**						
**CSR**						
H/Q Con 60°.s^−1^	0.49±0.1	0.51±0.1	0.01(−0.14 to 0.1)	0.51±0.1	0.51±0.07	0.005(−0.09 to 0.1)
H/Q Con 180°.s^−1^	0.47±0.2	0.50±0.2	0.03(−0.18 to 0.11)	0.51±0.1	0.52±0.1	0.001(−0.114 to 0.107)
H/Q Con 300°.s^−1^	0.68±0.3	0.61±0.1	−0.07(−0.11 to 0.26)	0.67±0.4	0.71±0.2	0.04(−0.36 to 0.28)
**FSR**						
F/S Quadriceps	0.48±0.1	0.50±0.1	0.02(−0.11 to 0.06)	0.46±0.1	0.48±0.05	0.02(−0.12 to 0.08)
F/S Hamstring	0.69±0.3	0.60±0.1	0.09(−0.18 to 0.37)	0.58±0.2	0.62±0.2	0.04(−0.27 to 0.19)
**DCR**						
H_ECC_/Q_CON_ 120°.s^−1^	0.69±0.2	0.64±0.2	−0.04(−0.13 to 0.21)	0.70±0.2	0.67±0.2	−0.02(−0.06 to 0.1)

**Legend:** CSR = Conventional strength ratio (concentric knee flexion/concentric knee extension); FSR = Fast/Slow speed ratio (net peak torque at 300°.s^−1^/net peak torque at 60°.s^−1^); DCR = dynamic control ratio (peak net eccentric hamstrings torque/peak net concentric quadriceps torque); Q = Quadriceps muscles; H = Hamstring muscles; con = concentric; Nm = Newton meter; °.s^−1^ = degree per second; * p<0.05; **p<0.01.

### DCR between Pre- and Post-tests

Paired *t*-test indicated significant differences in the dominant leg in the 11+ group (t = 2.68, p = 0.02). The results showed decrease in DCR in the dominant leg (p<0.05) by 40%. Paired *t*-test indicated significant differences in the non-dominant legs of the 11+ group (t = 3.87, p = 0.003). For the non-dominant leg, paired *t*-test indicated significant decrease in DCR (p<0.05) by 30%. However, no significant differences were shown in the HarmoKnee group (p>0.05).

### FSR between Pre- and Post-tests

The means of pre- and post-test values were analysed using paired *t*-test. In the 11+ group, significant increase was recorded by 8% only in the non-dominant leg between pre- and post-tests in the quadriceps (t = 2.37, p = 0.03). But no significant differences were shown in the HarmoKnee and control groups (p>0.05).

### Comparison of Variables between Groups

#### CSR between groups

The one-way ANCOVA indicated significant main effect between the 11+, HarmoKnee and control groups in the non-dominant leg at 60°.s^−1^ (F_2,32_ = 4.1, p = 0.02). The Bonferroni post-hoc test did not reveal significant differences between groups (p>0.05).

#### DCR between groups

For the DCR, the one-way ANCOVA indicated significant differences in the dominant leg (F_2,32_ = 3.6, p = 0.03) and non-dominant leg (F_2, 32_ = 10.9, p = 0.000). However, the Bonferroni post-hoc test revealed significant differences only in the non-dominant leg between the 11+ and control groups (p = 0.04) as well as the HarmoKnee and control groups (p = 0.04) (Table 4).

#### FSR between groups

The one-way ANCOVA indicated no significant differences for quadriceps muscles in the dominant leg (F_2,32_ = 0.21, p = 0.80) and non-dominant leg (F_2,32_ = 1.67, p = 0.20). The hamstring muscles revealed no significant differences in the dominant leg (F_2,32_ = 0.58, p = 0.56) and non-dominant leg (F_2,32_ = 0.63, p = 0.53).

### Comparison between Dominant and Non-dominant Legs

The paired *t*-test did not show significant differences between the dominant and non-dominant legs in the CSR at 60°.s^−1^, at 180°.s^−1^, at 300°.s^−1^, DCR and FSR in any of the groups.

## Discussion

The aim of this study was to investigate the effect of the FIFA 11+ and HarmoKnee on CSR, DCR and FSR in young male professional soccer players. The results of the present study showed that the CSR of the players after 8 weeks of training using the 11+ and HarmoKnee were lower than the average values at various angular speeds, below 0.61, 0.72, and 0.78 at 60°.s^−1^, 180°.s^−1^ and 300°.s^−1^. These drops in strength ratio of soccer players have been reported by previous researchers [11], [25], [32]. Similarly, this phenomenon is also seen in other sports. Pieter et al. [33] studied the isokinetic strength ratio of professional American martial arts athletes and found their CSRs were below average [33].

In the present study, the 11+ program significantly increased the CSR between pre- and post-test in the non-dominant leg at 60°.s^−1^ by 8%. The 11+ group also indicated significant differences in the dominant leg and non-dominant leg in DCR and increased by 8% for FSR in the quadriceps of the non-dominant leg. However, no significant differences were shown in the HarmoKnee group and control group when pre- and post-test data were compared. These results support the proposal that an 8-week FIFA 11+ program is superior in improving the strength ratio. These results are in agreement with Brito et al. [14]. They investigated the 11+ program on young male soccer players and reported significant increase in the CSR at 60°.s^−1^ by 14.8% and the DCR by 13.8% only in the non-dominant leg [14].

In the 11+ group, there was a significant increase between pre-test and post-test by 8% for FSR in the quadriceps of the non-dominant leg. However, no significant differences were shown between pre- and post-test in the HarmoKnee and control groups. Furthermore, the results did not indicate significant differences in FSR between the 11+, HarmoKnee and control groups in both legs and muscles. To our knowledge, there is only one study that investigated FSR on male soccer players. The study indicated no significant differences in FSR in the quadriceps and hamstrings of both legs in male soccer players after a 90-min soccer-specific intermittent program [25]. The present results support this proposal that more training elements should be added in both programs to increase the strength of the quadriceps and hamstrings.

Comparison between groups showed significant main effect in CSR only at 60°.s^−1^ in the non-dominant leg. The previous studies support the notion that this produces the highest torque at the slow speed (60°.s^−1^) [34]–[36]. The time available for contact between actin and myosin filaments decreases with increasing velocity of concentric activity (Huxley model); thus the period of the contact phase reduces in the overall cycle [32]. Cross-bridges have to be re-released shortly after their connection without enough time to produce power, so the proportion of combined bridges in the muscle declines, and produces lower strength [32], [37]. More motor units can be recruited at slower speeds than higher speeds allowing more torque production [38]. We believe that for a comprehensive assessment of strength ratio, measurement at slow angular velocity should be included since it produces the highest net peak torque.

In both experimental groups the DCR declined after the 8 weeks of training. This shows that in both groups the quadriceps strength increased more than that of the hamstring. In other words, certain components of the intervention programs have higher impact on the quadriceps’ isokinetic strength than on that of the hamstring. Further modification of both programs may be required to fully realise the normal DCR. The hamstring strength exercises, such as the Nordic hamstring [39]–[42], hamstring curl [39], single leg eccentric hamstring windmills and prone leg drops [40], which has been shown to increase hamstring muscle strength can be added to the programs [39]–[42]. The quadriceps strength has been shown to be highly correlated to ball kicking speed [6], hence it is advantageous to have a higher value. Nevertheless, a disproportionate increment in quadriceps strength against that of the hamstring will increase the anterior tibia shearing of ACL and also predispose to hamstring strain. It is imperative that more hamstring exercises are added to the 11+ and HarmoKnee programs to improve the functional balance of the players. The hamstring plays a protective role during extension of the knee particularly in terminal swing of a sprint [43].

The DCR of both groups was less than the normal average (<1.0). Athletes with DCR less than 1.0 when measured at higher velocities (>120°.s^−1^) are predisposed to knee injuries [33], [35]. DCR provides more suitable measure relating to knee function such as kicking, acceleration, deceleration etc. during play [12], [43]. The quadriceps strength would be more improved than that of the hamstring due to the nature of demands of running and kicking in soccer. Playing soccer requires activities such as kicking, jumping vertically and heading the ball in which there is weight-bearing in taking-off and landing. These demands may change the strength ratio in young professional soccer players [43]. Tourny-Chollet et al. [6] reported that the DCR of the soccer players is significantly lower than sedentary subjects at 60°.s^−1^ (0.80 vs. 0.93) and at 120°.s^−1^ (0.88 vs. 1.03) for both the dominant and non-dominant legs [6]. Therefore, the results of our study support the proposal that the young professional soccer players are exposed to higher knee injury risks than other sports. The knee injury risk in professional soccer is high and occurs frequently by non-contact mechanisms [44]. One of the causes for high knee injuries in professional soccer players is strength imbalance between hamstring and quadriceps.

It was found that there were no significant differences between the dominant and non-dominant legs in the strength ratios measured. Generally in soccer the dominant leg is used to kick the ball, while the non-dominant leg has the main role of providing postural support. This definition of footedness is commonly accepted by researchers [5], [45]. However, professional soccer players can perform kicking of the ball bilaterally and prefer to use both legs in different situations. This could be the possible cause of lack of differences observed between dominant and non-dominant legs in professional soccer players.

There is a limitation in this study that should be addressed. It must be noted that isokinetic dynamometer evaluates the net torque (output) of force of knee flexion and extension, not exactly the moment of hamstring or quadriceps muscles create.

### Conclusions

The main finding of this research is that there are different degrees of changes in CSR, DCR and FSR following the 11+ and HarmoKnee programs. The 11+ showed significant improvement in CSR and FSR, but the HarmoKnee program did not demonstrate any improvement. Interestingly CSR in both programs showed the greatest increase in strength at slow speed (60°.s^−1^) than at fast speed (300°.s^−1^). This study also revealed that the changes gained in all the three ratios following the 11+ and HarmoKnee programs did not differ between the dominant or non-dominant leg. Further emphasis on certain exercises to improve the ratios or new additions of exercises may help to enrich the two injury prevention programs.

### What does this Article Add?

Most professional young soccer players have higher predisposition of getting knee injuries. In comparison the FIFA 11+ program seems to show better improvement in CSR and FSR than the HarmoKnee program in the non-dominant leg. Both programs are lacking in exercise components that would improve hamstring strength which in turn would improve the balance in CSR and DCR. Measurement of CSR at low speed (60°.s^−1^) would allow a comprehensive assessment of CSR.
